# Insulin Promotes Glycogen Storage and Cell Proliferation in Primary Human Astrocytes

**DOI:** 10.1371/journal.pone.0021594

**Published:** 2011-06-27

**Authors:** Martin Heni, Anita M. Hennige, Andreas Peter, Dorothea Siegel-Axel, Anna-Maria Ordelheide, Norbert Krebs, Fausto Machicao, Andreas Fritsche, Hans-Ulrich Häring, Harald Staiger

**Affiliations:** Division of Endocrinology, Diabetology, Angiology, Nephrology and Clinical Chemistry, Department of Internal Medicine, Member of the German Center for Diabetes Research (DZD), Eberhard Karls University Tübingen, Tübingen, Germany; University of Bremen, Germany

## Abstract

**Introduction:**

In the human brain, there are at least as many astrocytes as neurons. Astrocytes are known to modulate neuronal function in several ways. Thus, they may also contribute to cerebral insulin actions. Therefore, we examined whether primary human astrocytes are insulin-responsive and whether their metabolic functions are affected by the hormone.

**Methods:**

Commercially available Normal Human Astrocytes were grown in the recommended medium. Major players in the insulin signaling pathway were detected by real-time RT-PCR and Western blotting. Phosphorylation events were detected by phospho-specific antibodies. Glucose uptake and glycogen synthesis were assessed using radio-labeled glucose. Glycogen content was assessed by histochemistry. Lactate levels were measured enzymatically. Cell proliferation was assessed by WST-1 assay.

**Results:**

We detected expression of key proteins for insulin signaling, such as insulin receptor β-subunit, insulin receptor substrat-1, Akt/protein kinase B and glycogen synthase kinase 3, in human astrocytes. Akt was phosphorylated and PI-3 kinase activity increased following insulin stimulation in a dose-dependent manner. Neither increased glucose uptake nor lactate secretion after insulin stimulation could be evidenced in this cell type. However, we found increased insulin-dependent glucose incorporation into glycogen. Furthermore, cell numbers increased dose-dependently upon insulin treatment.

**Discussion:**

This study demonstrated that human astrocytes are insulin-responsive at the molecular level. We identified glycogen synthesis and cell proliferation as biological responses of insulin signaling in these brain cells. Hence, this cell type may contribute to the effects of insulin in the human brain.

## Introduction

It was known for many years, that the insulin receptor is widely expressed throughout the central nervous system (CNS) [1]. Despite that, the brain was still considered to be a mostly insulin-independent organ, since glucose uptake is not significantly stimulated by insulin [2]. However, in the last years, evidence for an important role of this hormone in various brain functions emerged [3], [4]. Among others, central insulin actions were found to be involved in the regulation of body weight and food intake [4], in the processing of food-related stimuli [5] as well as in memory [6]. Many of these studies were done *in vivo* without knowing the responsible cell type for insulin's actions; most of the *in vitro* work focused on specific neuron subpopulations.

However, besides neurons, there are many other cell types within the brain that may potentially contribute to the function of the whole organ [7]. Indeed, there are at least as many glial cells as neurons [8]. Among these, astrocytes are very interesting from a metabolic point of view: they take up glucose and store energy as glycogen. Even if the astrocytes' glycogen content is low compared to ‘classical’ glycogen storage organs such as liver and skeletal muscle, it is of great importance for neuronal function [9].

Further on, astrocytes release lactate, which may be taken up by neurons as an energy source in times of need, i.e. during neuronal activity or in hypoglycemia [9]–[11]. However, there is still debate about the significance of this lactate shuttling from astrocytes towards neurons *in vivo* [11]–[13].

Astrocytes are also part of the blood-brain barrier [14] and exert a pivotal role in the regulation of cerebral blood flow and thus contribute to the regulation of neurons' supply of nutrients and oxygen [15].

Furthermore, these cells take up and release various neurotransmitters, thereby modulating and terminating the action of transmitters secreted from neurons or communicating with other cells [16].

More globally, astrocytes were recently shown to be involved in a brain function that is also influenced by insulin: memory formation [17].

On a cellular level, astrocyte-enriched cultures from rodents were shown to form glycogen after stimulation with very high concentrations of insulin [18]–[20]. Literature on the issue whether insulin additionally stimulates glucose uptake into astrocytes is inconsistent [20], [21]. Furthermore, increased cell growth in response to high insulin levels was demonstrated in rodent astrocyte-enriched cultures [19]. Recently, a possible role of the insulin signaling cascade for the regulation of glutamate transporter 1, an important molecule for glutamate uptake into astrocytes, has been demonstrated [22].

However, most of these functions were detected in rodent astrocytes. Recently, fundamental differences between rodent and human astrocytes were reported (e.g. in size, structural complexity and diversity, kinetics of activation) [23]. Oberheim et al. suggested that these differences between astrocytes from rodent vs. human origin may even explain some of the general differences between mice and men [23]. Thus, findings in rodent astrocytes can not generally be transferred to humans.

The aim of this study was, hence, to analyze in human primary astrocytes whether insulin signaling occurs, and if so, whether metabolic functions are influenced by the hormone.

## Results

**Figure 1:**
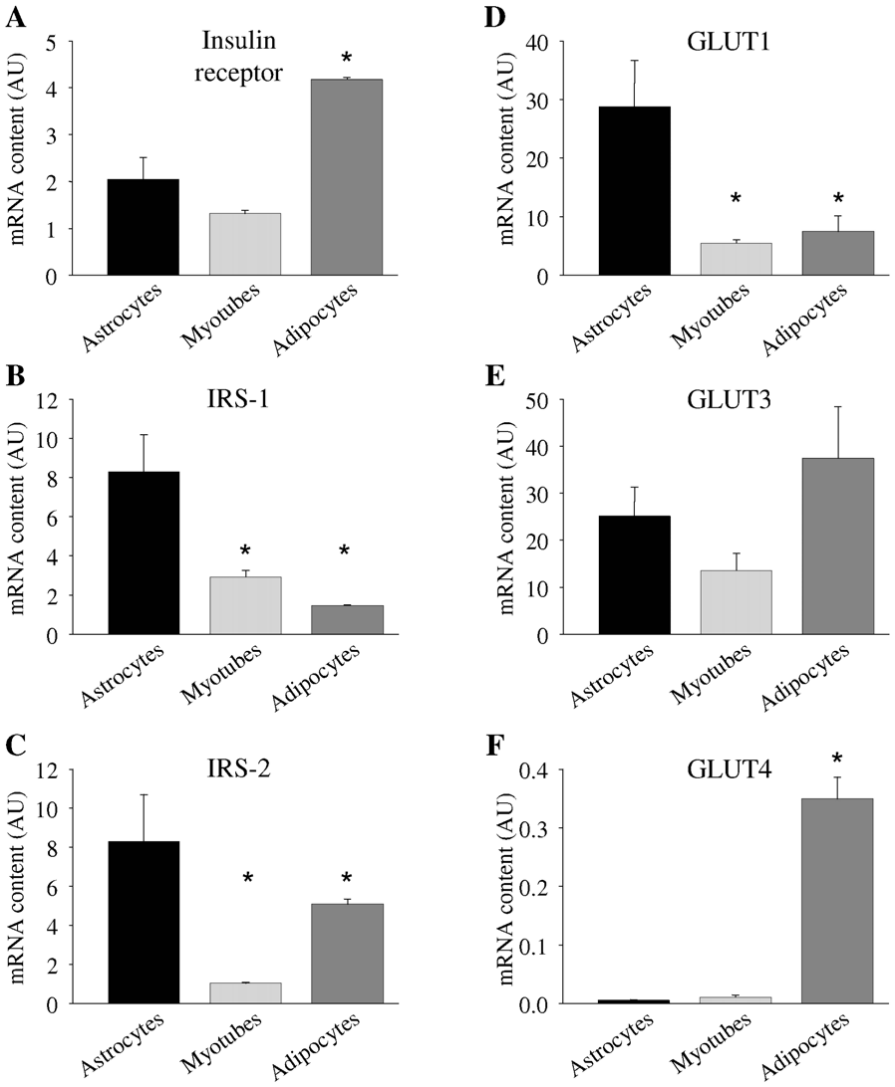
mRNA expression in human astrocytes (black bars) in comparison to human myotubes (light grey bars) and human adipocytes (dark grey bars). (**A**) Insulin receptor, (**B**) IRS-1, (**C**) IRS-2, (**D**) GLUT1, (**E**) GLUT3, (**F**) GLUT4. mRNA expression was normalized for mRNA of the housekeeping gene *Rps13*. Bars represent means + SEM. N = 3. There were significant differences between the groups in all mRNA expressions analyzed (ANOVA, all p≤0.0254) except for GLUT3 mRNA (ANOVA, p = 0.2). * indicates significant difference from astrocytes (Tukey Kramer post hoc test p<0.05).

**Figure 2:**
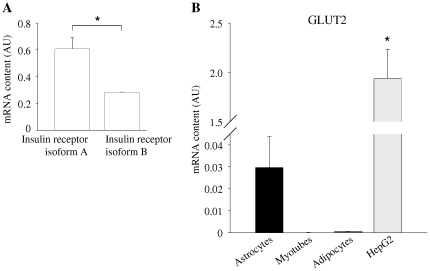
mRNA expression of insulin receptor isoforms A and B as well as GLUT 2 in human astrocytes. (**A**) mRNA expression of insulin receptor isoform A and B in human astrocytes. Given are means + SEM, N = 3. *  =  there were significant differences between groups (Student's t-test, p = 0.0181). (**B**) mRNA expression of GLUT2 in human astrocytes (black bar), human myotubes (white bar), human adipocytes (dark grey bar), and HepG2 (human hepatocellular carcinoma cell line, light grey bar). mRNA expression was normalized for mRNA of the housekeeping gene *Rps13*. Bars represent means + SEM. N = 3. There were significant differences between the groups (ANOVA, p = 0.0006). * indicates significant difference from astrocytes (Tukey Kramer post hoc test p<0.05).

First, we examined the mRNA expression of major insulin signaling molecules in human astrocytes in comparison to human myotubes and human adipocytes: Astrocytes express insulin receptor in similar amounts as myotubes (figure 1 A). Roughly, two thirds of astrocytes' insulin receptors are isoform A and around one third is isoform B (figure 2 A). Expression levels of insulin receptor substrate (IRS)-1 as well as IRS-2 were significantly higher in astrocytes than in the other cell types (figures 1 B and C). Glucose transporter (GLUT) 1 mRNA was found in significantly greater amounts in astrocytes than in the other analyzed cells (figure 1 D), while GLUT3 expression was in a comparable range in all three cell types (figure 1 E). Nominally, more GLUT2 mRNA than in adipocytes or myotubes was present in astrocytes, but around 70-fold less than that found in HepG2 cells (figure 2 B). GLUT4 was barely detectable in astrocytes and myotubes compared to adipocytes (figure 1 F).

**Figure 3:**
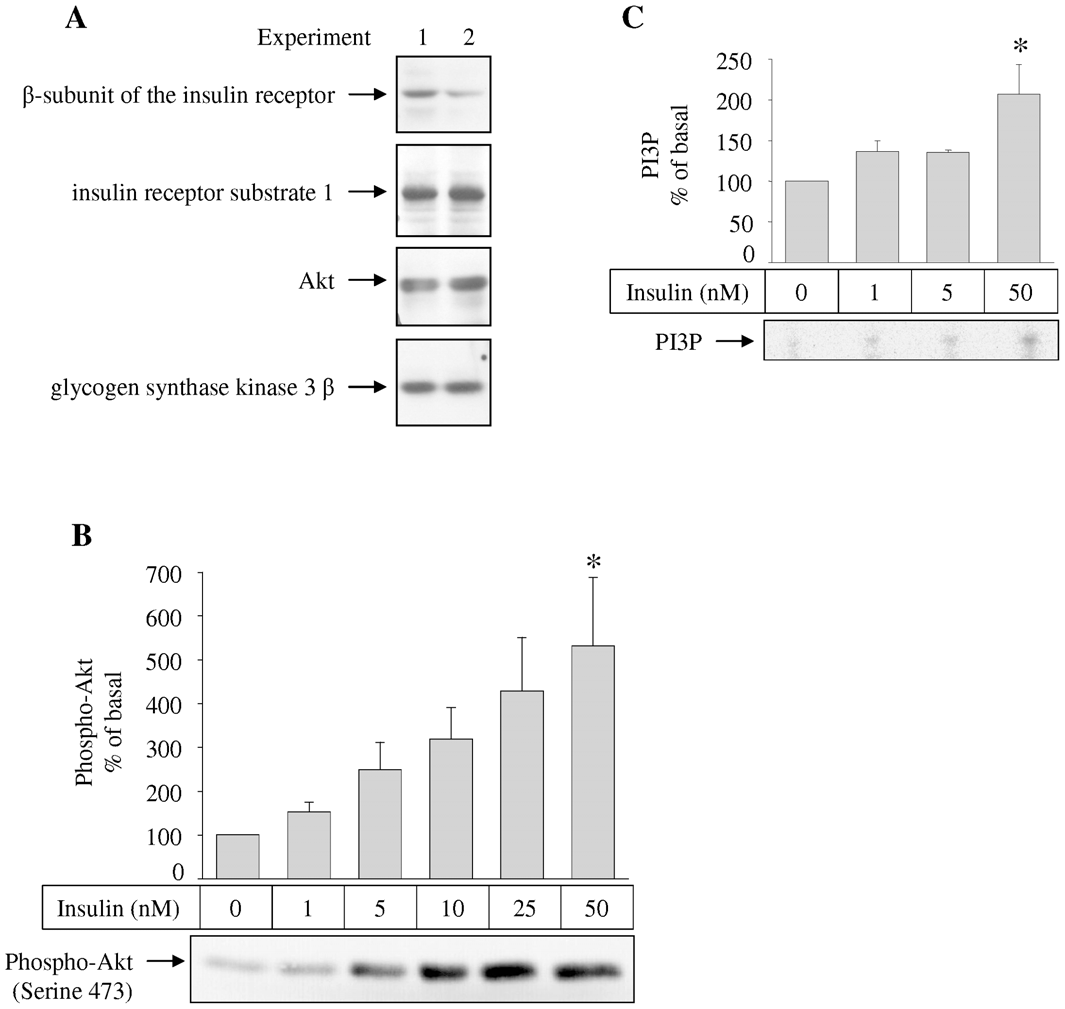
Protein expression, insulin-stimulated Akt phosphorlyation, and insulin-stimulated phosphatidylinositiol 3′-phosphorylation in human astrocytes. (**A**) Equal amounts of total cell lysates of two independent experiments were loaded onto a SDS-PAGE gel. The indicated proteins were detected using specific antibodies. (**B**) Cells were stimulated with the indicated insulin concentrations for 15 minutes and lysed afterwards. Akt phosphorylation was detected by a phospho-specific antibody. A representative western blot is shown in the lower part. Bars in the diagram represent means + SEM. N = 6. There were significant differences between the groups (ANOVA, p = 0.0108). * indicates significant difference from 0 nM insulin (Tukey Kramer post hoc test p<0.05). (**C**) Following insulin stimulation with insulin for 30 minutes, cells were harvested, and lysates were immunoprecipitated with anti IRS-1 antibodies and a PI-3 kinase assay was performed using L-α-phosphatidylinositol as substrate. A representative image is shown in the lower part. Bars in the diagram represent means + SEM. N = 3. There were significant differences between the groups (ANOVA, p = 0.0267). * indicates significant difference from 0 nM insulin (Tukey Kramer post hoc test p<0.05).

At the protein level, we detected insulin receptor's β-subunit, IRS-1, Akt, and glycogen synthase kinase (GSK) 3 β in astrocytes (figure 3 A).

To investigate, if the insulin-signaling cascade is functional and can thus be activated in human astrocytes, we performed a PI-3 kinase assay. Following insulin stimulation, we found increased phosphorylation of L-α-phosphatidylinositol towards phosphatidylinositol-3′-phosphate indicating increased PI-3 kinase activity. The insulin effect was dose-dependent and could be detected at concentrations as low as 1 nM (figure 3 C). Accordingly, Akt phosphorylation on serine 473 was increased by insulin stimulation, starting at concentration of 1 nM (figure 3 B). Increase in Akt phosphorylation and PI-3 kinase activity reached statistical significance at 50 nM (figure 3 B and C).

**Figure 4:**
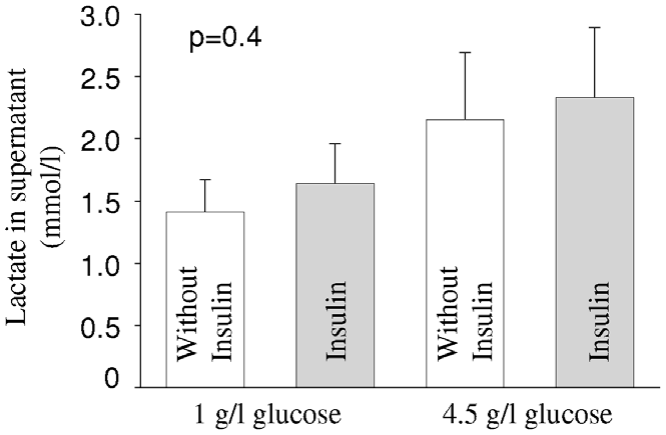
Effects of insulin stimulation and glucose concentration on lactate secretion of human astrocytes. Prior to experiment, NHAs were either starved in medium containing 1 g/l glucose or kept in medium with 4.5 g/l. Medium was than replaced by new medium with the same glucose concentrations without (white bars) or with 50 nM insulin (grey bars). After 8 hours, lactate concentration in the supernatant was measured. Bars represent means of five independent experiments + SEM. There were no significant differences between groups (ANOVA, p = 0.4).

Further on, we studied, whether metabolic functions of human astrocytes are influenced by insulin: glucose uptake into cells was not affected by treatment with either 50 or 100 nM of insulin for 15 minutes (ANOVA p = 0.7, N = 5). Lactate content of the supernatants was neither influenced by stimulation with 50 nM insulin for 8 hours nor by variation of glucose concentration (figure 4).

**Figure 5:**
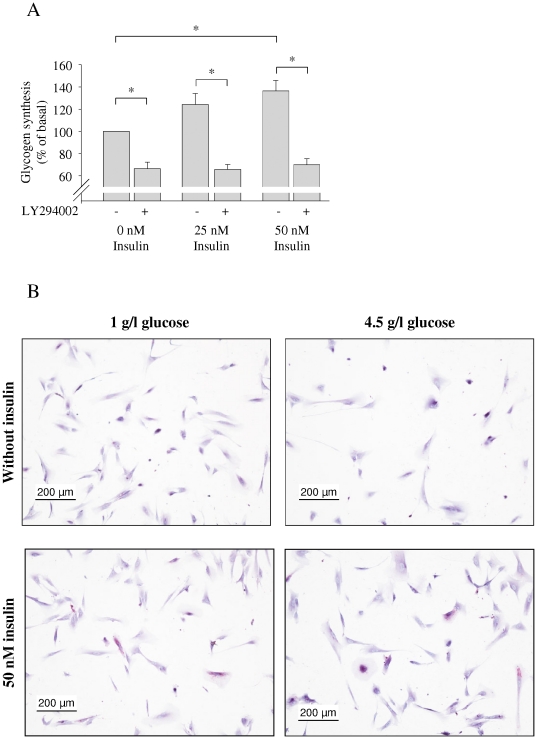
Effects of insulin stimulation on glycogen synthesis (A) and glycogen content (B) of human astrocytes. (**A**) The indicated cells were preincubated with the PI-3 kinase inhibitor LY294002 for 1 hour. Cells were stimulated with the indicated concentrations of insulin for 3 hours. Glycogen synthesis in the absence of insulin and LY294002 was set as 100%. Bars represent means of at five independent experiments + SEM. There were significant differences between the groups (ANOVA, p<0.0001). * indicates significant difference (Tukey Kramer post hoc test p<0.05). (**B**) Prior to experiment, NHAs were either starved in medium containing 1 g/l glucose (left lane) or kept in medium with 4.5 g/l (right lane). Medium was than replaced by new medium with the same glucose concentrations without (upper panels) or with 50 nM insulin (lower panels). After three hours of stimulation, cells were stained for glycogen (pink). Shown are representative examples of at least three independent experiments.

However, when analyzing incorporation of labeled glucose into glycogen, we detected a significant increase following insulin stimulation. To test if this occurs via the classical PI-3 kinase/Akt pathway, we added the PI-3 kinase inhibitor LY294002 and found a significant decrease of glucose incorporation into glycogen of around 30% under basal conditions, and insulin-stimulated glycogen synthesis was abolished when LY294002 was added (figure 5 A). Thus, basal glycogen synthesis as well as the insulin effect on glycogen synthesis are PI-3 kinase-dependent.

We furthermore stained NHA cells for glycogen content before and after stimulation with 50 nM insulin for 3 h under two different glucose concentrations, 1 g/l and 4.5 g/l. Whilst barely any of the unstimulated cells contained detectable amounts of glycogen, some of the insulin-stimulated cells stained positive for glycogen (figure 5 B). There were no detectable differences between the glucose concentrations, neither in the stimulated nor in the unstimulated cells (figure 5 B).

**Figure 6:**
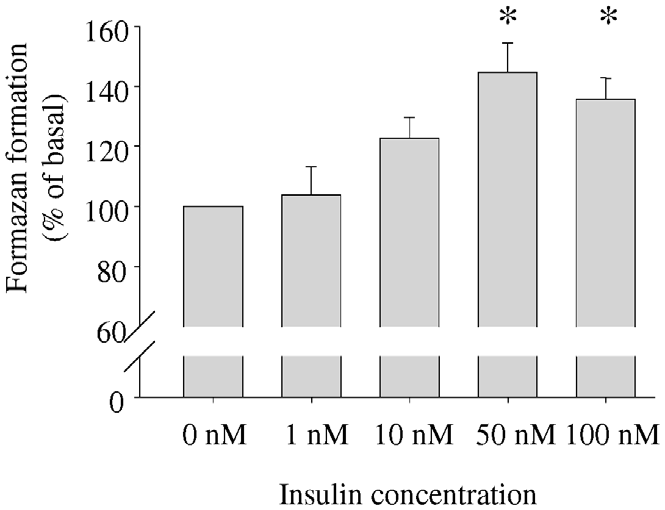
Effects of insulin stimulation on human astrocytes' proliferation. After starvation for at least 24 hours, cells were treated with the indicated concentrations of insulin for 3 days. Thereafter, cell proliferation was assessed by WST-1 assay. Bars represent means of six independent experiments + SEM. There were significant differences between the groups (ANOVA, p = 0.0007). * indicates significant difference from 0 nM (Tukey Kramer post hoc test p<0.05).

Finally, we investigated cell proliferation following insulin treatment for three days by WST-1 assay. With increasing insulin dose, there was an increase in dye formation that reached statistical significance at a concentration of 50 nM (figure 6), indicating cell proliferation. The morphology of the NHA cells did not appear different between the insulin concentrations.

## Discussion

In the present study, we could clearly demonstrate the presence of major insulin signal transduction molecules in human primary astrocytes. In addition, the insulin signaling cascade was shown to be functionally active in these cells. We found insulin to stimulate glycogen formation and cell proliferation, while glucose uptake and lactate secretion were unaffected by the hormone.

Insulin receptor was present in comparable amounts as in the other two tested insulin-sensitive cell types. Around two-thirds of the insulin receptors in astrocytes were the receptor's isoform A. The expression of this isoform A in the human brain is well known [24]. In contrast to isoform B, this isoform has, besides its binding capacity for insulin, a high affinity for insulin-like growth factor 2 (IGF-2) [24]. Substantial amounts of IGF-2 are present in various regions of the human brain [25] – effects on astrocytes have not been studied in detail, yet. Furthermore, isoform A was found to confer mitogenic responses [26]. In agreement, insulin induced proliferation of astrocytes in our experiments.

Downstream of the insulin receptor, we detected both, IRS-1 and IRS-2 in human astrocytes. Both of them are important for insulin signaling within the brain: Genetic variation within the *IRS-1* locus was shown to determine insulin responsiveness of the human brain [27] and partially dysregulated IRS-2 signaling causes hyperphagia and obesity in animals [28].

For the glucose transporters, human astrocytes showed high GLUT1 and GLUT3 expressions, while only very little GLUT2 and almost no GLUT4 was detected. The first-mentioned two transporters are insulin-independent, while GLUT4 is regulated by insulin [29]. The expression pattern, hence, explains why we could not detect any effect of insulin stimulation on astrocytes' glucose uptake.

Even if expressed nearly ubiquitous [29], GLUT1 is believed to be responsible for glucose transport across the blood-brain-barrier [30], a structure to which astrocytes contribute [14], [15]. The importance of this transporter within the brain is underlined by rare genetic defects within this gene causing cerebral damages [31].

While in cultured rat astrocytes GLUT3 expression was only detectable after pretreatment with endotoxin or hypoxia [32], we detected GLUT3 in primary human astrocytes even under basal conditions. Since this transporter has a high glucose affinity [29], it might possibly serve as the major glucose transporter in human astrocytes.

In this study, we demonstrated the insulin signaling cascade to be functional in terms of increased PI-3 kinase activity and Akt Serine 473 phosphorylation in these cells. Increments in PI-3 kinase activity were already detected at an insulin concentration as low as 1 nM, concentrations that are commonly exceeded in the blood of healthy humans after food intake. Even if insulin concentrations in the cerebrospinal fluid are markedly lower than those in the plasma [33], astrocytes might be exposed to comparable concentrations *in vivo* due to their close contact with blood vessels [14], [15].

After characterizing human astrocytes as an insulin-responsive cell type, we investigated whether these cells' metabolic functions are influenced by the hormone: similar to other glycogen-storing cell types like hepatocytes or muscle cells, we found increased glycogen storage in astrocytes following insulin stimulation. This energy storage is important to support neurons with energy [10], since neurons can not store glycogen for themselves. But neuronal activity triggers the mobilization of astrocytes' glycogen [34], probably via the release of neurotransmitters [35]. This energy support is necessary especially during intense neuronal activity [9]–[11], [36]. Thus, larger glycogen stores in astrocytes due to insulin action increase the rapidly available amount of energy and thus might be permissive for stimulation of neuronal activation. Thereby insulin-stimulated glycogen storage in astrocytes could possibly contribute to insulin-mediated changes within the brain.

Surprisingly, when adding the PI-3-kinase inhibitor LY294002, there was a marked decrease in glycogen synthesis even without stimulation of the insulin signaling cascade. This points to an endogenous activity of the PI-3-kinase/Akt pathway under basal conditions that can, however, still be enhanced by insulin. Whether basal, non-insulin-stimulated glycogen formation is further regulated, and if so, what pathways or stimuli are involved has not been studied yet.

By contrast to rodent astrocyte-enriched cultures, we did not detect increment in lactate secretion after insulin stimulation, neither under high nor under low glucose. Maybe the presence of neurons is necessary to allow insulin to stimulate lactate secretion in human astrocytes. To further investigate this question, experiments in co-cultures of astrocytes and neurons are necessary.

We found cell numbers to increase after adding increasing insulin concentrations in the medium for three days. This is in accordance to previous findings in rodent astrocytes [19]. In humans, astrocytes are among the brain cell types possessing the ability to proliferate in adults. It is known that astrocyte numbers increase rapidly as part of the reaction called astrogliosis (e.g. during inflammation or tissue damage [37], [38]). However, by light microscopy we did not detect morphologic characteristics of astrogliosis [38], i.e. hypertrophy of the cell body or processes. The insulin stimulated increase in cell numbers may thus be gliogenesis [39], another type of astrocyte proliferation that takes place without those morphological changes. Gliogenesis can occur in the adult brain and is stimulated by various cytokines [39]. Therefore, insulin may be another inductor of astrocyte proliferation in the human brain. Whether astrocyte numbers are higher in persons with higher insulin levels (e.g. in obesity) has not been studied. Our results point towards this possibility. Thus, this would be worth to be further investigated especially because differences in cerebral functions between lean and obese persons have been found [27], but only little about underlying cellular mechanisms has been revealed.

In conclusion, we characterized human astrocytes as an insulin-responsive cell type in terms of glycogen formation and cell proliferation. Hence, this cell type might contribute to the effects of insulin in the human brain.

## Methods

### Cell culture

Normal Human Astrocytes (NHA) derived from fetal human brain are commercially available as cryopreserved, primary-derived cultures (Lonza, Basel, Switzerland). These cells are guaranteed to stain positive for GFAP (Glial Fibrillary Acid Protein), one marker for astrocytes. NHA were grown in AGM medium (Lonza) containing 3% fetal bovine serum, 4.5% glucose, and reagents from BulletKits (Lonza) in a humidified incubator at 37°C and 5% CO_2_. Prior to each experiment, cells were washed twice with PBS (Lonza) and starved in DMEM (1 g/l glucose, Lonza) +0.5% FCS for 48 h. The FCS used for our experiments contains 54.9 ng/ml IGF-1. Since our starvation medium contains 0.5% FCS this results in a concentration of 0.27 ng/ml (0.04 nmol/l) in the starvation medium. For insulin stimulation, human recombinant insulin was used (Novo Nordisk, Bagsvard, Denmark).

Human myotubes and adipocytes were grown from primary precursor cells and differentiated *in vitro* as described earlier [40], [41]. Human HepG2 hepatoma cells were grown in MEM medium containing 10% FCS and 2 mM Glutamine until 80% confluence.

### Western blot

The cells were lysed in lysis buffer containing 1% Triton X-100, 50 mM HEPES, pH 7.5, 10% glycerol, 150 mM NaCl, 1.5 mM EGTA, 10 mM sodium pyrophosphate, 100 mM NaF, 2 mM sodium orthovanadate, 10 µg/ml aprotinin, 1 mM phenylmethylsulfonylfluoride. Lysates were cleared by centrifugation at 13,000 *g* for 15 minutes at 4°C. After lysis, protein content was quantified by Bradford's method. Equal amounts of protein were loaded on each lane of a SDS-PAGE (sodium dodecyl sulfate polyacrylamid gel electrophoresis) gel. After run, proteins were transferred to a nitrocellulose membrane (Schleicher & Schuell, Dassel, Germany), and incubated with the primary antibody mentioned below for one hour. After three washes, the membranes were incubated with secondary horseradish peroxidase-conjugated goat anti-mouse or goat anti-rabbit antibodies (Sigma-Aldrich, St. Louis, MO, USA). After three washes, detection was performed using the ECL system (Amersham Life Science, Braunschweig, Germany). For quantification of signal intensity of Western blots, the EasyWin32 Herolab Software was used.

The following antibodies were used: Insulin receptor β-subunit polyclonal antibodies from own production (detecting KKN GRI LTL PRS NPS); IRS-1 antibodies (Millipore, Billerica, MA, USA); Pan-Akt antibodies (R&D Systems, Minneapolis, MN, USA); GSK3β antibodies (Cell Signaling, Danvers, MA, USA); Phospho-Akt (Serine 473, Cell Signaling, Danvers, MA, USA).

*Staining* Glycogen was detected using the periodic acid-Schiff (PAS) reaction. Mayer's hemalum solution was used as a counterstain.

### Glucose uptake

Cells were incubated in Krebs-Ringer-HEPES buffer for 3 hours. Afterwards, they were stimulated with the indicated insulin concentrations for 15 minutes. Following stimulation, a mixture of 2-deoxy-[1-^3^H]-D-glucose (0.4 µCi/well) and 0.1 M non-labeled 2-deoxyglucose was added for three minutes. The reaction was stopped by washing with Krebs-Ringer-HEPES buffer twice. Then, cells were lysed and analyzed with a beta-counter.

### Lactate secretion

Cells were either starved in DMEM, 0.5% FCS, 1 g/l glucose or kept in DMEM, 0.5% FCS, 4.5 g/l glucose for 48 h. Afterwards, cells were stimulated with 50 nM of insulin for 48 hours or kept in medium without insulin as a control. Lactate was quantified in the supernatants using the lactate oxidase method with a commercial colorimetric assay for ADVIA 1800 Clinical Chemistry System (Siemens Medical Solutions, Eschborn, Germany).

### PI-3 kinase assay

Lysates of normal human astrocytes were immunoprecipitated with IRS-1 antibodies (Millipore, Billerica, MA, USA) and protein A-coupled sepharose. After washing, the precipitates were incubated with 0.1 mg/ml L-α-phosphatidylinositol (Sigma Aldrich, St. Louis, MO, USA) and 50 µM [γ^32^P]-adenosinetriphosphate (Perkin Elmer, Waltham, MA, USA) at room temperature for 10 minutes. After adding 150 µl 1 M HCl, lipids were extracted using 450 µl chloroform/methanol (1∶1) twice. Products were separated by thin layer chromatography. ^32^P-labeled phospholipids were detected by autoradiography.

### Glycogen synthesis

Cells were washed with pre-warmed PBS buffer twice and then incubated in DMEM (0.5% FCS, 1 g/l glucose) at 37°C for 3 hours. Afterwards, they were stimulated with the indicated insulin concentrations in DMEM medium containing ^14^C-D-glucose (0.6 µCi/well; GE Healthcare, Little Chalfont, UK). After three hours supernatant was discarded and the cells were washed two times with ice-cold PBS. Afterwards, 500 µl KOH (30%) was added onto the cells for 30 minutes at room temperature, until cells were detached. Precipitation solution was than mixed with 1 mg of glycogen and boiled for 30 minutes at 95°C. Samples were washed with ice-cold ethanol twice. Glycogen pellets were resuspended in H_2_O and analyzed with a beta-counter.

### Quantitative real-time RT-PCR

Cells were washed and harvested by trypsinisation. Cells were lysed with RLT and homogenized using QIAshredder (Qiagen, Hilden, Germany). Total-RNA was isolated using RNeasy columns (Qiagen), treated with RNase-free DNase I and transcripted to cDNA using Transciptior First Strand cDNA Synthesis kit (Roche Diagnostics, Mannheim, Germany). PCRs (in duplicates) were performed on a LightCycler 480 (Roche Diagnostics) using Probes Master and fluorescent probes from the Universal Probe Library (Roche Diagnostics). The following real-time PCR protocol was used: denaturation program (95°C for 5 minutes), an amplification and quantification program repeated 45 times (95°C for 10 seconds, 60°C for 30 seconds, 72°C for 1 second [fluorescence acquisition]), and finally a cooling down program to 4°C. Primers were designed using the Roche Probe Design 2 software (Roche Diagnositcs) and purchased from TIB MOLBIOL (Berlin, Germany).

Insulin receptor isoform A was amplified using the following primers: *forward* TTT CGT CCC CAG AAA AAC CTC T, *reverse* CCACCGTCACATTCCCAAC. Insulin receptor isoform B was amplified using primers: *forward* TTT TCG TCC CCA GGC CAT, *reverse* CCA CCG TCA CAT TCC CAA C. Both reactions used 5′ 6-FAM phosphoramidite-TCG CCA AGG GAC CTG CGT T-BBQ (4,4-Bis-[2-butyloctyloxy]-p-quaterphenyl) as a probe.

The other reactions used standard Roche probes and the following primers: Insulin receptor substrate (IRS)-1 *forward* GCC TAT GCC AGC ATC AGT TT, *reverse* TTG CTG AGG TCA TTT AGG TCT TC; IRS-2 *forward* TGA CTT CTT GTC CCA CCA CTT, *reverse* CAT CCT GGT GAT AAA GCC AGA; insulin receptor *forward* GCT GGA TTA TTG CCT CAA AGG, *reverse* TGA GAA TCT TCA GAC TCG AAT GG; glucose transporter (GLUT) 1 *forward* GGT TGT GCC ATA CTC ATG ACC, *reverse* CAG ATA GGA CAT CCA GGG TAG C; GLUT2 *forward* TGG TTT TCA CTG CTG TCT CTG, *reverse* CAT TCC AAT TAG AAA GAG AGA ACG TC; GLUT3 *forward* GCC CTG AAA GTC CCA GAT TT, *reverse* TTC ATC TCC TGG ATG TCT TGG; GLUT4 *forward* CTG TGC CAT CCT GAT GAC TG, *reverse* CGT AGC TCA TGG CTG GAA CT, RPS13 *forward* CCC CAC TTG GTT GAA GTT GA, *reverse* ACA CCA TGT GAA TCT CTC AGG A.

All RNA data is presented relative to the housekeeping gene *RPS13* using the ΔΔCt method.

### Cell proliferation assay

The water soluble tetrazolium (WST)-1 assay was used to estimate astrocytes cell numbers according to the manufacturer's instructions (Roche Molecular Biochemicals, Mannheim, Germany). The amount of formazan dye formed directly correlates to the number of metabolically active, viable cells. Equal amounts of NHA were seeded in each well of a 96-well plate. After starvation for at least 24 hours, cells were treated with insulin for 3 days.

### Statistical analyses

For all statistical analyses, the software package JMP 8.0.2 (SAS Institute, Cary, NC, USA) was used. Two-group comparisons were performed using Student's t-test. Differences between multiple groups were tested by ANOVA. Tukey–Kramer test was used as a post-hoc test. Results with values of p≤0.05 were considered statistically significant.
